# 

**Published:** 1883-09

**Authors:** 


SEPTEMBER, 1883.

NEW SERIES
Vol. II—No. 12

### ATLANTA
### MEDICAL REGISTER.

(*ATLANTA MEDICAL AND SURGICAL JOURNAL.*)

EDITED BY

J. H. LOGAN, A.M., M.D.

H. H. DICKSON, PUBLISHER. TERMS, $2.50 A YEAR IN ADVANCE

ATLANTA, GEORGIA:

*H. H. DICKSON, BOOK AND JOB PRINTER AND BOOKBINDER.*

1883.

